# Photoinduced Long-Distance Hydrogen-Atom Transfer in Molecules with a 7-Hydroxyquinoline Frame and a Carbaldehyde or Aldoxime Group as the Intramolecular Hydrogen Transporting Crane

**DOI:** 10.1021/acs.jpca.3c00170

**Published:** 2023-04-03

**Authors:** Leszek Lapinski, Hanna Rostkowska, Jacek Nowacki, Maciej J. Nowak

**Affiliations:** Institute of Physics, Polish Academy of Sciences, Al. Lotników 32/46, 02-668 Warsaw, Poland

## Abstract

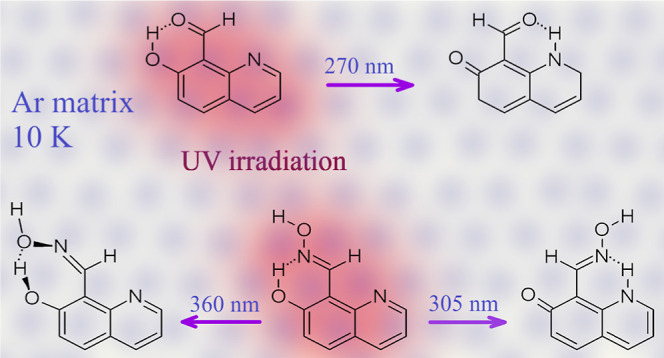

The photochemical properties of monomeric 7-hydroxyquinoline substituted at position 8 with carbaldehyde or aldoxime groups were studied for the molecules isolated in solid Ar low-temperature matrices (at 10 K). It was experimentally demonstrated that upon UV excitation, both carbaldehyde and aldoxime groups act as intramolecular cranes transmitting hydrogen atoms from the hydroxyl group to the remote nitrogen atom of the quinoline ring. Furthermore, in the case of 7-hydroxyquinoline-8-aldoxime (and its derivatives), the second photochemical channel was activated upon UV (λ > 360 nm) excitation. This process involves syn–anti isomerization around the double C=N bond in the aldoxime group. The structures of the reactant hydroxy tautomeric form and the photoproduced isomers of the studied molecules were unequivocally determined by means of IR spectroscopy combined with theoretical predictions of the IR spectra of the candidate structures.

## 1 Introduction

**Scheme 1:**
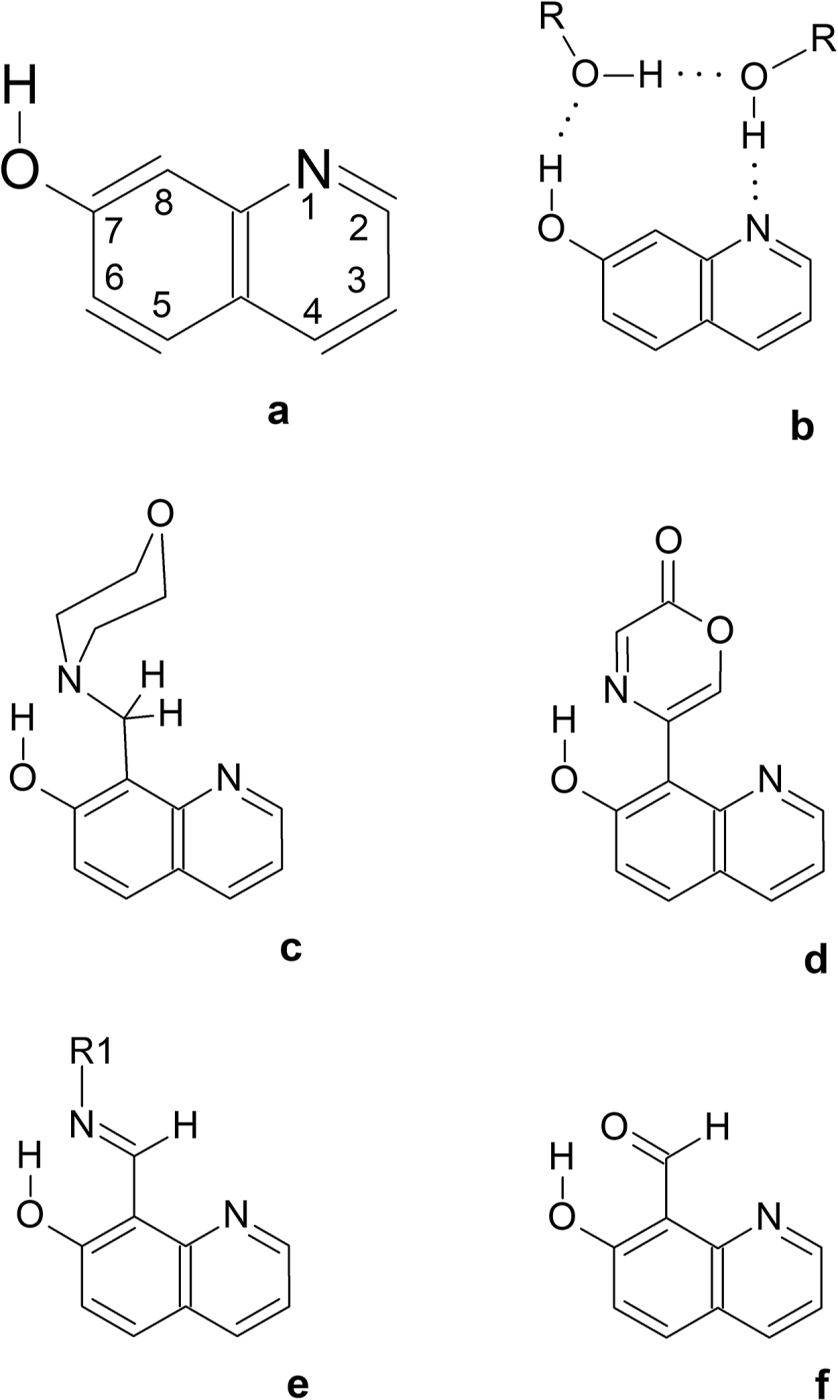
Molecular Structures of (a) 7-Hydroxyquinoline; (b) 7-Hydroxyquinoline and Two Protic Solvent Molecules; (c) 7-Hydroxy-8-(morpholinomethyl)-quinoline; (d) 7-Hydroxy-8-(oxazine-2-one)-quinoline; (e) R1 = Alkyl or Aryl, 7-Hydroxyquinoline-8-Schiff Base; R1 = OH, 7-Hydroxyquinoline-8-aldoxime; R1 = Acylhydrazone, 7-Hydroxyquinoline-8-acylhydrazone; (f) 7-Hydroxyquinoline-8-carbaldehyde

The change of the tautomeric form of 7-hydroxyquinoline (Scheme 1a) should involve a long-distance transfer of a hydrogen atom from the exocyclic oxygen atom to the endocyclic nitrogen atom. On the potential-energy surface of 7-hydroxyquinoline, the minima of the hydroxy and oxo tautomeric forms are separated by a very high barrier.^1^ Hence, in the ground electronic state of monomeric 7-hydroxyquinoline, a strictly intramolecular change of tautomeric form is not possible. For the compound dissolved in protic media (Scheme 1b), a solvent-assisted change of the tautomeric form occurs via consecutive proton transfers along the chain of hydrogen bonds connecting the proton-donating hydroxyl group and proton-accepting endocyclic nitrogen atom.^1−5^ In 7-hydroxyquinoline cyclic dimers, the temperature-induced change of the tautomeric form proceeds by a concerted transfer of two protons along −O–H···N hydrogen bonds.^6^ Nevertheless, for 7-hydroxyquinoline in protic solvents as well as for 7-hydroxyquinoline dimers, it is impossible to induce a long-lasting shift of the ratio of tautomer populations using methods such as excitation with UV light. Due to a very fast transfer of protons along the hydrogen bonds, the system returns instantaneously back to the tautomeric equilibrium existing before UV excitation.

Jalink et al.^7^ postulated that a group (such as the morpholinomethyl moiety) attached at position 8 to the 7-hydroxyquinoline frame can serve as a molecular crane allowing an intramolecular hydrogen-atom transfer to proceed in a UV-excited molecule such as 7-hydroxy-8-(morpholinomethyl)-quinoline (Scheme 1c). With this intramolecular crane, hydrogen atom initially attached to the exocyclic oxygen atom of the O–H group can be transferred to the endocyclic nitrogen atom. The other bistable molecular system with a 7-hydroxyquinoline frame and an oxazine-2-one crane (7-hydroxy-8-(oxazine-2-one)-quinoline, Scheme 1d) has been proposed by Sobolewski.^8^ Recently, 7-hydroxyquinoline Schiff bases (Scheme 1e),^9^ 7-hydroxyquinoline-8-acylhydrazone,^10^ and 7-hydroxyquinoline with a sulfonamide group^11^ have been considered as candidates for bistable tautomeric switches. Molecules with a 7-hydroxyquinoline frame and the carbaldehyde group (Scheme 1f), the simplest moiety that can play the role of the intramolecular crane, have also been investigated.^12,13^ In all the molecules mentioned above, the crane fragment contains a nitrogen or oxygen heteroatom placed in such a position that it serves as an electron-density donor in a hydrogen bond with the O–H group of the 7-hydroxyquinoline frame (see Scheme 1c–f). This intramolecular hydrogen bond is relatively strong because it closes a six-membered quasi-ring. In the relaxed structure of such six-membered quasi-rings, no intramolecular strain^14^ weakens the interactions of the hydrogen atom with the heteroatoms involved.

The theoretical model of intramolecular long-distance hydrogen-atom-transfer processes occurring in compounds with the 7-hydroxyquinoline frame and a crane group containing a heteroatom has been presented by Sobolewski.^8,15^ According to this model, the first step that follows the UV excitation of the molecule is the well-known excited-state intramolecular proton transfer (ESIPT) process.^16−20^ As a result of this process, a hydrogen atom is translocated from the O–H group to the heteroatom of the crane moiety. In the excited state of the molecule with the hydrogen atom attached to the crane fragment, the C–C bond (connecting the crane group with the 7-hydroxyquinoline frame) gains the double (or partially double) character. This gives rise to the “ethylene-like” twisting^21^ of the crane fragment around the C–C axle with respect to the plane defined by the quinoline rings. Then, during further relaxation, the hydrogen atom can be “dropped” on the nitrogen atom of the quinoline frame. This latter step completes the ESIPT-driven hydrogen-atom transfer from the exocyclic O–H group to the remote endocyclic N atom. In principle, such a hydrogen-atom-transfer process should be photoreversible.^8,12^

Most of the experimental investigations of the UV-induced hydrogen-atom-transfer processes in compounds with a 7-hydroxyquinoline frame and crane moiety (morpholinomethyl, carbaldehyde, or Schiff base) have been conducted using UV–vis absorption and UV-induced fluorescence spectroscopy.^7,9,13,22^ Double fluorescence, typical of the compounds with intramolecular hydrogen bonds, was observed for these species dissolved, for example, in acetonitrile. Alongside these two luminescence bands, a third band was usually observed as a shoulder on the long-wavelength wing of the red-shifted band ascribed to the ESIPT-type fluorescence. This third band was tentatively interpreted as an indication of the tautomer photogenerated in the long-distance hydrogen-atom-transfer process. Unfortunately, broad and vibrationally unresolved fluorescence spectra do not provide any direct information about the structure of the photogenerated species. Hence, such spectra cannot serve as a basis for reliable identification of the photoproduced tautomer with the hydrogen atom transferred to the endocyclic nitrogen atom. In order to get a better insight into the phototautomeric processes occurring in UV-excited 7-hydroxy-8-(morpholinomethyl)-quinoline dissolved in deuterated acetonitrile, van der Loop et al.^23^ carried out a series of UV–pump–IR-probe experiments. In these experiments, the compound was excited with a very short UV pulse and, thereafter, time-resolved vibrational spectra were recorded (in the 1650–1410 cm^–1^ range) for different delay times. As a result, three or four vibrational features were observed, which support (especially that at ca 1640 cm^–1^) identification of the photoproduced N–H tautomer. According to the conducted measurements, the lifetime of this photoproduct in acetonitrile solution is ca. 40 ns.

The current work is devoted to investigations of UV-induced transformations of 7-hydroxyquinoline derivatives with carbaldehyde or aldoxime groups attached at position 8 of the quinoline frame. The molecules were studied using the matrix-isolation technique. Due to low temperature and an inert solid Ar medium, this technique allows (i) minimization of the environmental effects on the studied phototransformations so that the observed photoprocesses are governed only by intrinsic properties of the molecule and (ii) stabilization of the photoproduced species. Usually, the lifetime of photoproducts generated in solid-noble-gas matrices is so long that it allows the characterization of the photoproducts with stationary spectroscopic techniques. Based on such spectral characterization, a reliable identification of the matrix-isolated products can be obtained. Application of the matrix-isolation method gave a big advantage in the studies of photoinduced long-range hydrogen-atom transfer and other isomerization processes occurring in the studied 7-hydroxyquinoline derivatives.

## 2 Experimental Section

7-Hydroxyquinoline-8-carbaldehyde was synthesized by the Reimer–Tiemann reaction according to the procedures described in refs (9) and (12). 7-Hydroxy-2-methylquinoline-8-carbaldehyde and 7-hydroxy-2-methoxy-4-methylquinoline-8-carbaldehyde were obtained using analogous procedures. 7-Hydroxyquinoline-8-aldoxime, 7-hydroxy-2-methylquinoline-8-aldoxime, and 7-hydroxy-2-phenylquinoline-8-aldoxime were synthesized by reactions of the corresponding 7-hydroxyquinoline-8-carbaldehyde derivatives with hydroxylamine.

Low-temperature matrices were prepared according to the procedures described in detail elsewhere.^24^ Infrared spectra of matrix-isolated species were collected (with 0.5 cm^–1^ resolution) using a Thermo Nicolet iS50R FTIR spectrometer. The matrix-isolated compounds were irradiated with UV light. In most experiments, an HBO 200 high-pressure mercury lamp fitted with a water filter and an appropriate Schott cutoff filter transmitting light with λ > 270 nm, λ > 295 nm, λ > 320 nm, λ > 335 nm, or λ > 360 nm was used as a source of UV light. In some cases, the matrices were exposed to quasimonochromatic (FWHM = 15 nm) UV light (λ_max_ = 278 nm) or (λ_max_ = 305 nm) emitted by 6060 LG Innotek LEDs.

## 3 Computational Section

The Gaussian 09, revision D.01, program^25^ was used to calculate, within the harmonic approximation, the infrared spectra of the molecules considered in the current work. The computations were carried out at the DFT(B3LYP)/6-311++G(d,p) level.^26−28^ The calculated harmonic wavenumbers were scaled by a factor of 0.98.

## 4 Results

### 4.1 Photochemical Behavior of Matrix-Isolated 7-Hydroxyquinoline with No Substituent at the C8 Atom

**Figure 1:**
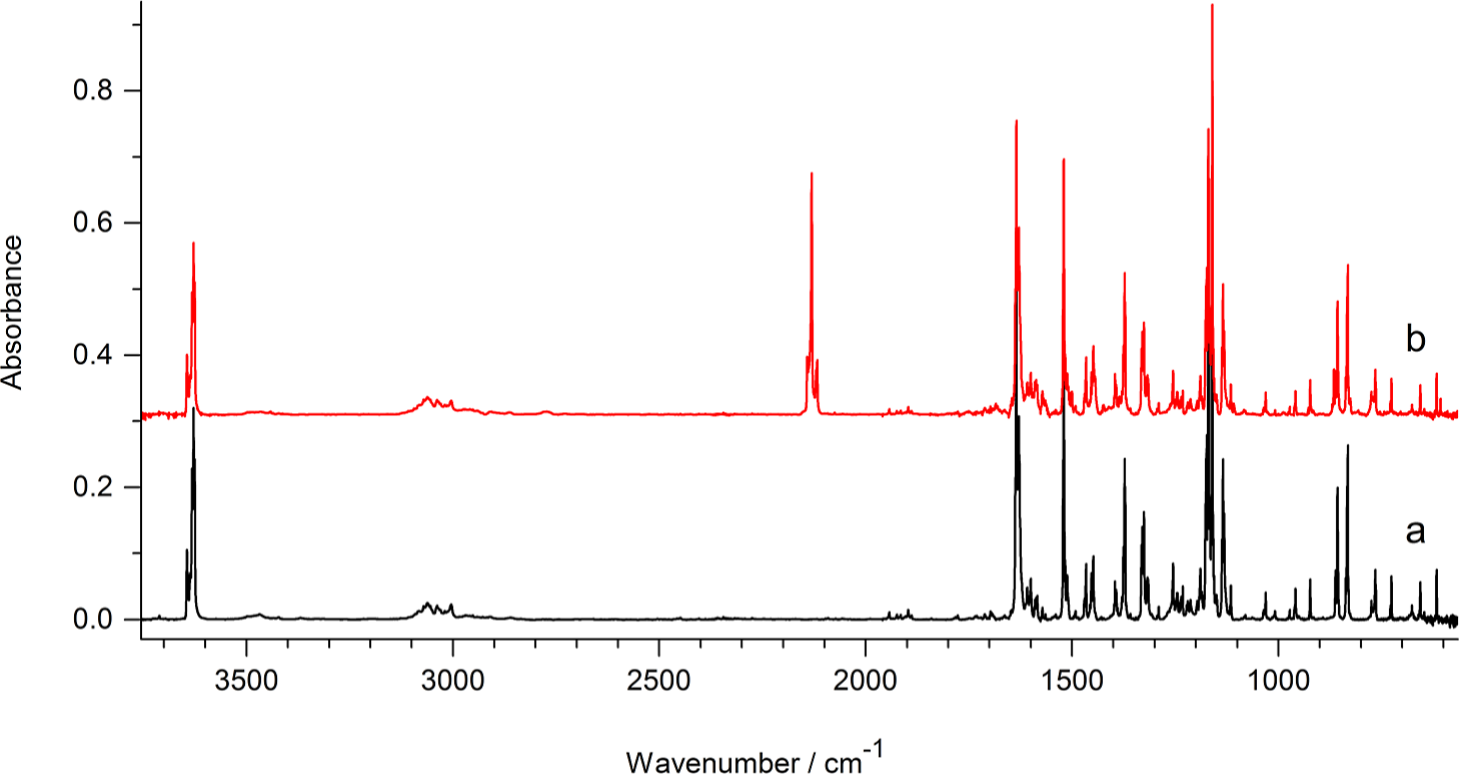
Infrared spectra of 7-hydroxyquinoline isolated in an argon matrix: (a) recorded after deposition of the matrix; (b) recorded after 5 h of UV (λ = 278 nm) irradiation.

7-Hydroxyquinoline molecules isolated in Ar matrices exclusively adopt the hydroxy tautomeric form (Scheme 1a). In the infrared spectrum of the matrix-isolated compound, the band due to the stretching vibration of the O–H group appears at 3628 cm^–1^ with lower-intensity sub-bands at 3644, 3632, and 3626 cm^–1^ (Figure 1a). In this spectrum, there are no IR absorptions in the range of 3460–3360 cm^–1^ where a band due to the stretching vibration of the N1–H group in the oxo form of 7-hydroxyquinoline might be expected.

Irradiation of matrix-isolated 7-hydroxyquinoline with UV (λ = 278 nm) light led to slow consumption of the hydroxy form of the compound and generation of the ketene product (Figure 1b). The ketene product was generated not only upon irradiation at λ = 278 nm but also upon UV irradiation at longer wavelengths: λ > 320 nm or λ = 305 nm. Following the interpretation given by Sekine et al.,^29^ the photoproduction of the ketene species was proven by the appearance of the strong band at 2131 cm^–1^ (with sub-bands at 2141 and 2117 cm^–1^) in the spectrum recorded after UV irradiation of matrix-isolated 7-hydroxyquinoline (Figure 1b).

During UV irradiation of 7-hydroxyquinoline isolated in Ar matrices, the hydroxy → oxo phototautomeric process (involving a long-distance hydrogen-atom transfer) either does not occur at all or it occurs with extremely low efficiency. In the spectra recorded after UV irradiation of matrix-isolated 7-hydroxyquinoline monomers, no new IR bands appear (even low-intensity bands), which could be reliably identified as spectral indications of the oxo photoproduct.

### 4.2 Long-Distance Hydrogen-Atom Transfer in Molecules with the 7-Hydroxyquinoline Frame and the Carbaldehyde Group as an Intramolecular Crane

**Scheme 2:**
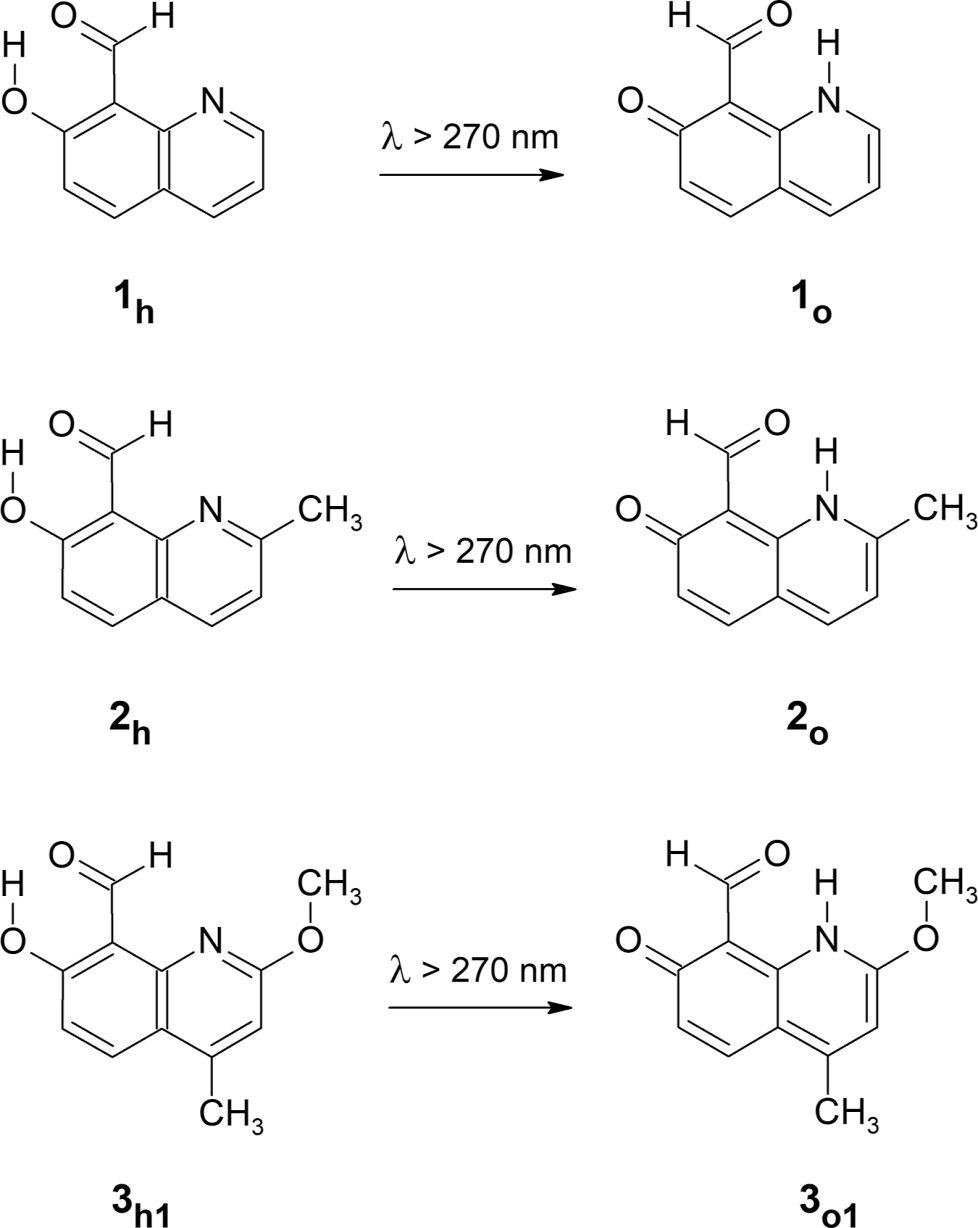
UV-Induced Hydrogen-Atom-Transfer Processes in 7-Hydroxyquinoline-8-carbaldehyde (**1**); 7-Hydroxy-2-methylquinoline-8-carbaldehyde (**2**); and 7-Hydroxy-2-methoxy-4-methylquinoline-8-carbaldehyde (**3**)

Molecules of 7-hydroxyquinoline-8-carbaldehyde (**1**) and related compounds such as 7-hydroxy-2-methylquinoline-8-carbaldehyde (**2**) and 7-hydroxy-2-methoxy-4-methylquinoline-8-carbaldehyde (**3**) isolated in low-temperature argon matrices exclusively adopt their most stable hydroxy tautomeric form (**1**_**h**_, **2**_**h**_, and **3**_**h**_, respectively, Scheme 2). This is confirmed by the good agreement between the experimental infrared spectra of the matrix-isolated species and the spectra computed for the respective hydroxy tautomers (Figures S1–S3 in the Supporting Information).

In the structures of the hydroxy forms of the compounds, the O–H group at position 7 is involved in an intramolecular hydrogen bond with the C=O fragment of the carbaldehyde substituent. This interaction strongly affects the properties of 7-hydroxyquinolines with the carbaldehyde group at position 8. In the higher-wavenumber regions of the infrared spectra of matrix-isolated monomers of these compounds, the strength of the −O–H···O=C– interaction is reflected by the large red shift and very pronounced broadening of the νOH bands, with respect to the position and the shape of the band due to free OH in 7-hydroxyquinoline (Figure S4a–c, in the Supporting Information).

**Figure 2:**
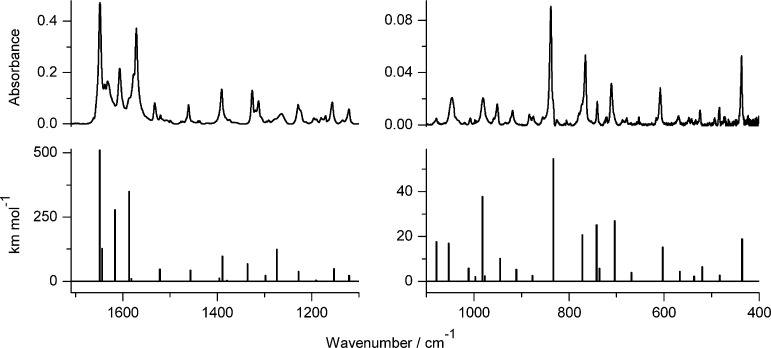
Infrared spectrum of photoproduct(s) generated upon 140 min of UV (λ > 270 nm) irradiation of 7-hydroxyquinoline-8-carbaldehyde (**1**) isolated in an Ar matrix, compared with the theoretical spectrum, calculated at the DFT(B3LYP)/6-311++G(d,p) level, for the oxo tautomer **1**_**o**_ of the compound (Scheme 2).

The effects of irradiation of 7-hydroxyquinoline-8-carbaldehyde (**1**) isolated in an Ar matrix with UV light are different from the effects of UV irradiation of matrix-isolated 7-hydroxyquinoline. For **1**, only a very low quantity of the ketene product (revealing itself by the appearance of very low-intensity bands at 2139 and 2158 cm^–1^) was generated upon λ > 320 nm irradiation. The major product of UV-induced conversions of matrix-isolated **1** is the oxo form (**1**_**o**_) of the compound with the hydrogen atom transferred from the O–H group to the N1 nitrogen atom (Scheme 2). Similarity of the experimentally collected spectrum of the photoproduct generated upon UV (λ > 270 nm) irradiation of 7-hydroxyquinoline-8-carbaldehyde monomers and the spectrum theoretically calculated for the oxo form (**1**_**o**_) strongly supports this identification (Figure 2).

**Figure 3:**
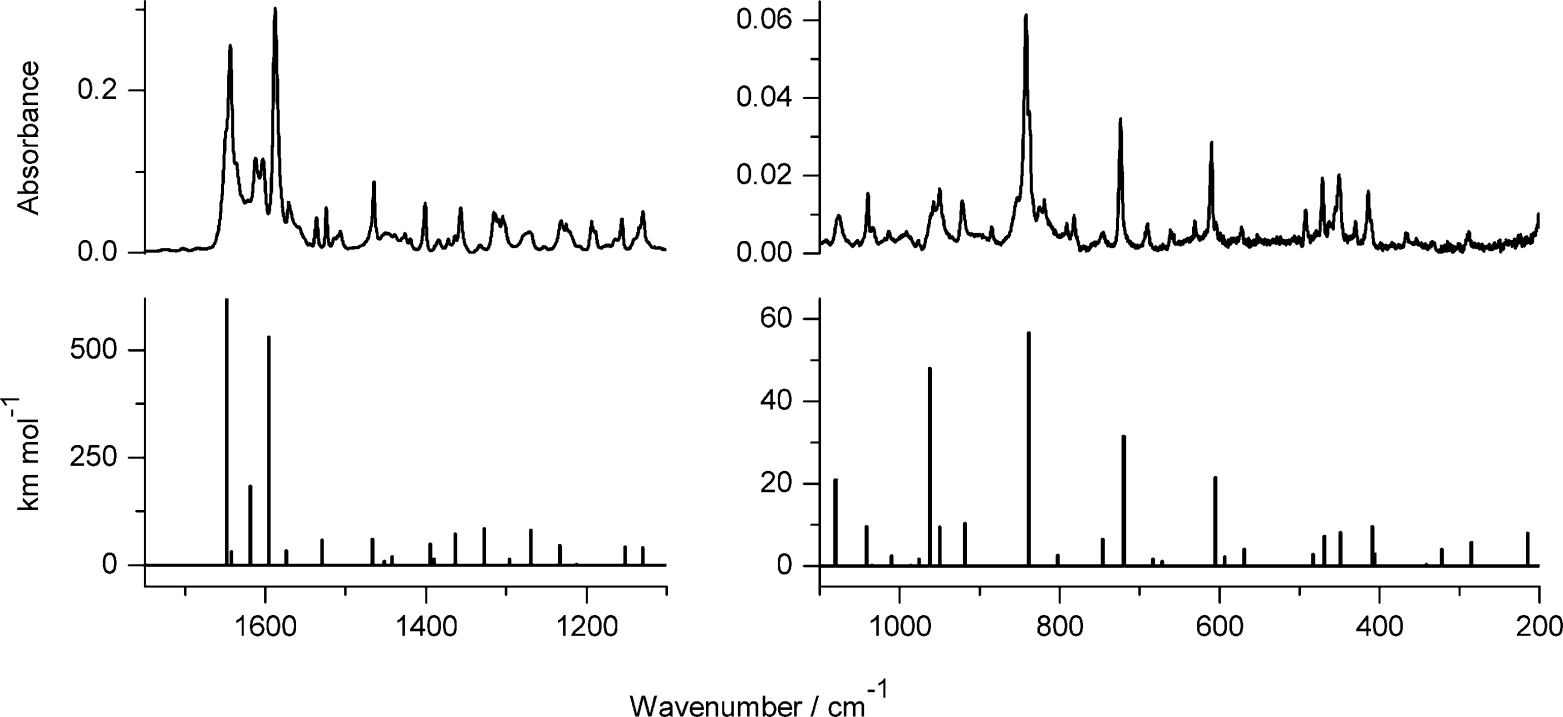
Infrared spectrum of photoproduct(s) generated upon 100 min of UV (λ > 270 nm) irradiation of 7-hydroxy-2-methylquinoline-8-carbaldehyde (**2**) isolated in an Ar matrix, compared with theoretical spectrum, calculated at the DFT(B3LYP)/6-311++G(d,p) level, for the oxo tautomer **2**_**o**_ of the compound (Scheme 2).

For 7-hydroxyquinoline-8-carbaldehyde substituted with a methyl group at position 2 of the quinoline frame, that is, for 7-hydroxy-2-methylquinoline-8-carbaldehyde (**2** in Scheme 2), photoprocesses induced by exposure to UV (λ > 270 nm) light were also strongly dominated by hydrogen-atom transfer from the O–H group to the N1 atom. The photoproduct was identified by comparison (Figure 3) of its experimental IR spectrum with the spectrum computed for the oxo tautomer (**2**_**o**_).

We want to emphasize that although the patterns of the bands in the experimental IR spectra of the photoproducts generated upon UV excitation of 7-hydroxyquinoline-8-carbaldehyde, 7-hydroxy-4-methylquinoline-8-carbaldehyde,^12^ and 7-hydroxy-2-methylquinoline-8-carbaldehyde are different for each of the compounds, the theoretical calculations carried out for the oxo tautomers reproduce these spectral patterns very well in every case. This clearly demonstrates that the assignment of the photoproduct structure is not based on the accidental similarity of the experimental and theoretically predicted spectra. Hence, the UV-induced long-distance hydrogen-atom transfer in 7-hydroxyquinoline-8-carbaldehyde and its derivatives may be treated as a very well-documented photochemical process.

Photochemical behavior has been also studied for 7-hydroxy-2-methoxy-4-methylquinoline-8-carbaldehyde (**3** in Scheme 2) isolated in Ar matrices. For this compound, the experimental picture may be somewhat complicated because of the presence of the flexible methoxyl substituent. The −O–CH_3_ group can be directed either toward the N1 nitrogen atom (**3**_**h1**_ in Scheme 2) or to the C3 carbon atom of the quinoline frame (**3**_**h2**_ in Figure S3 in the Supporting Information). According to the DFT(B3LYP)/6-311++G(d,p) calculations, carried out for the compound in the initial hydroxy form, the first of these conformers **3**_**h1**_ is significantly (by 22 kJ mol^–1^) more stable. The juxtaposition of the experimental spectrum with the spectra simulated for both conformers of the hydroxy form (Figure S3 in the Supporting Information) shows that the lower-energy conformer **3**_**h1**_ was trapped from the gas phase into an Ar matrix.

**Figure 4:**
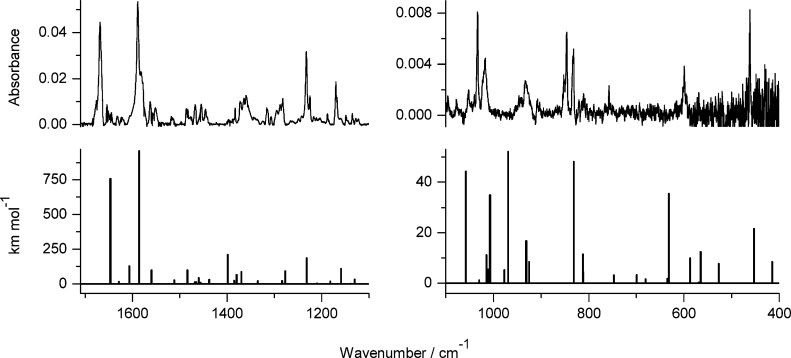
Infrared spectrum of photoproduct(s) generated upon 140 min of UV (λ > 270 nm) irradiation of 7-hydroxy-2-methoxy-4-methylquinoline-8-carbaldehyde (**3**) isolated in an Ar matrix, compared with theoretical spectrum, calculated at the DFT(B3LYP)/6-311++G(d,p) level, for the oxo tautomer **3**_**o1**_ of the compound (Scheme 2).

The analogous oxo conformer (**3**_**o1**_ with the −O–CH_3_ group directed toward the N1 nitrogen atom, Scheme 2) seems to be the main product generated upon UV (λ > 270 nm) irradiation (Figure 4) of an Ar matrix containing monomers of 7-hydroxy-2-methoxy-4-methylquinoline-8-carbaldehyde. This conclusion is confirmed by a fairly good agreement between the experimental spectrum and the theoretical spectrum computed for **3**_**o1**_. This agreement is visibly better than that obtained for the other oxo conformer **3**_**o2**_, with the −O–CH_3_ group oriented toward the C3 atom (Figure S5 in the Supporting Information).

### 4.3 Long-Distance Hydrogen-Atom Transfer in Molecules with the 7-Hydroxyquinoline Frame and the Aldoxime Group as an Intramolecular Crane

In the preceding Section 4.2, the occurrence of long-distance hydrogen-atom transfer in compounds with the 7-hydroxyquinoline frame and the carbaldehyde group attached at position 8 was well documented. One can imagine that other small groups containing heteroatoms could also serve as a crane transporting hydrogen atom from the O–H group attached at position 7 to the remote N1 atom of the quinoline frame (Scheme 1). The aldoxime moiety is one of the candidate groups which may be tested.

**Scheme 3:**
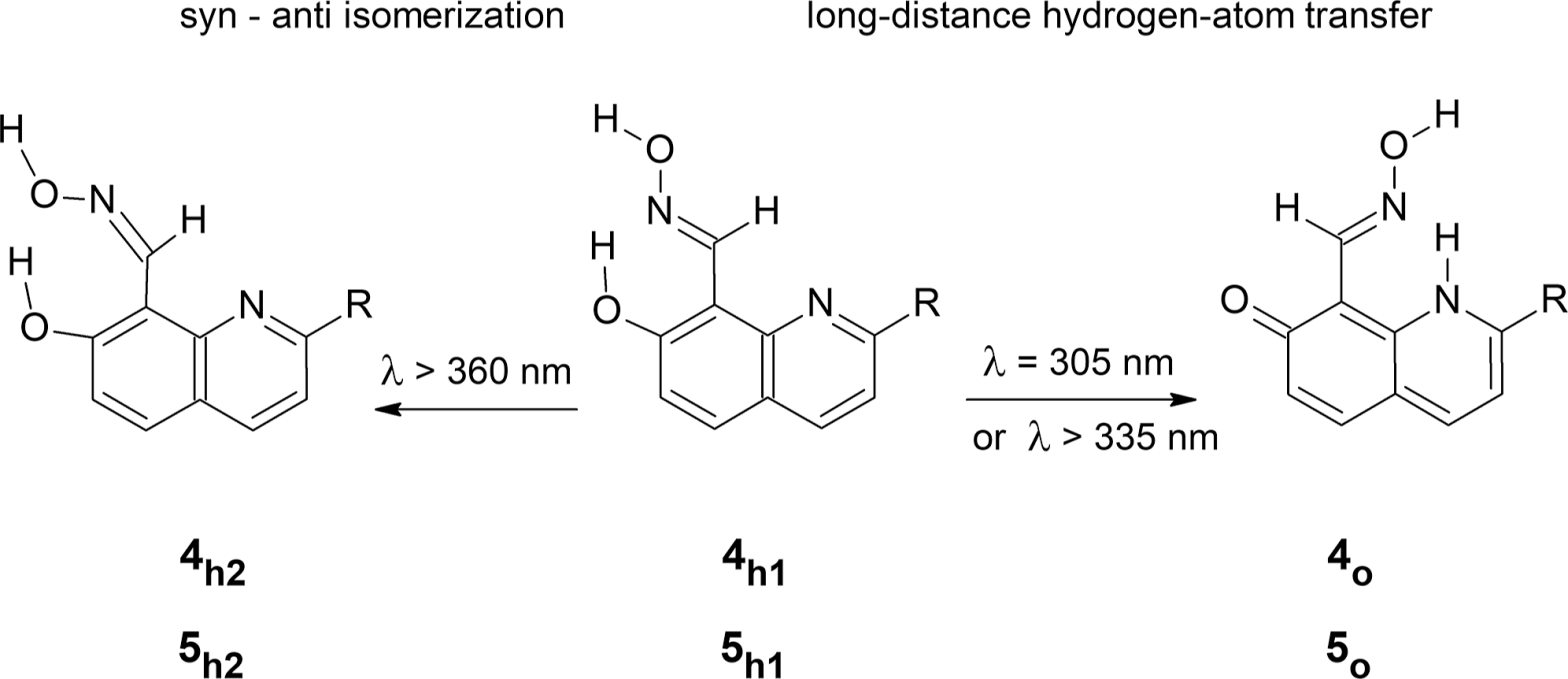
UV-Induced Processes Occurring in 7-Hydroxyquinoline-8-aldoxime (**4**, R = H) and 7-Hydroxy-2-methylquinoline-8-aldoxime (**5**, R = CH_3_)

The molecule of 7-hydroxyquinoline-8-aldoxime (**4** in Scheme 3) is built of the 7-hydroxyquinoline frame and an aldoxime −C(H)=N–O–H group attached at position 8. When trapped in a low-temperature Ar matrix, molecules of the compound adopt the most stable hydroxy form (**4**_**h1**_). It was confirmed by a good agreement between the experimental IR spectrum and the spectrum theoretically simulated for **4**_**h1**_ (Figure S6 in the Supporting Information). In the hydroxy form of the compound, an intramolecular hydrogen bond closes a six-membered ring involving the O–H group attached at position 7 of the quinoline frame and the N atom of the aldoxime fragment. Because the C7–O–H group participates in the hydrogen bond, the band due to the stretching vibration of this O–H group does not manifest itself as a well-defined, sharp absorption feature. Only the significantly red-shifted, very broad spectral structures (at wavenumbers 3100–2900 cm^–1^) can be interpreted as spectral signatures of this O–H stretching band (Figure S4d, in the Supporting Information).

The O–H group of the aldoxime fragment is not involved in any hydrogen bond. Hence, a sharp band originating from the stretching vibration of this O–H group is expected in the high-wavenumber region of the IR spectrum. In the experimental spectrum of **4** isolated in an Ar matrix, the band due to the stretching vibration of the O–H group from the aldoxime fragment was found at 3622 cm^–1^ (Figure S4d, in the Supporting Information).

For 7-hydroxyquinoline-8-aldoxime (**4**), not only the photoinduced long-distance hydrogen-atom transfer, which was the strongly dominating photoprocess in the case of UV-irradiated 7-hydroxyquinoline-8-carbaldehyde (**1**), but also the UV-induced syn–anti isomerization within the aldoxime −C(H)=N–O–H group (Scheme 3) need to be considered as possible phototransformations.^30−32^

**Figure 5:**
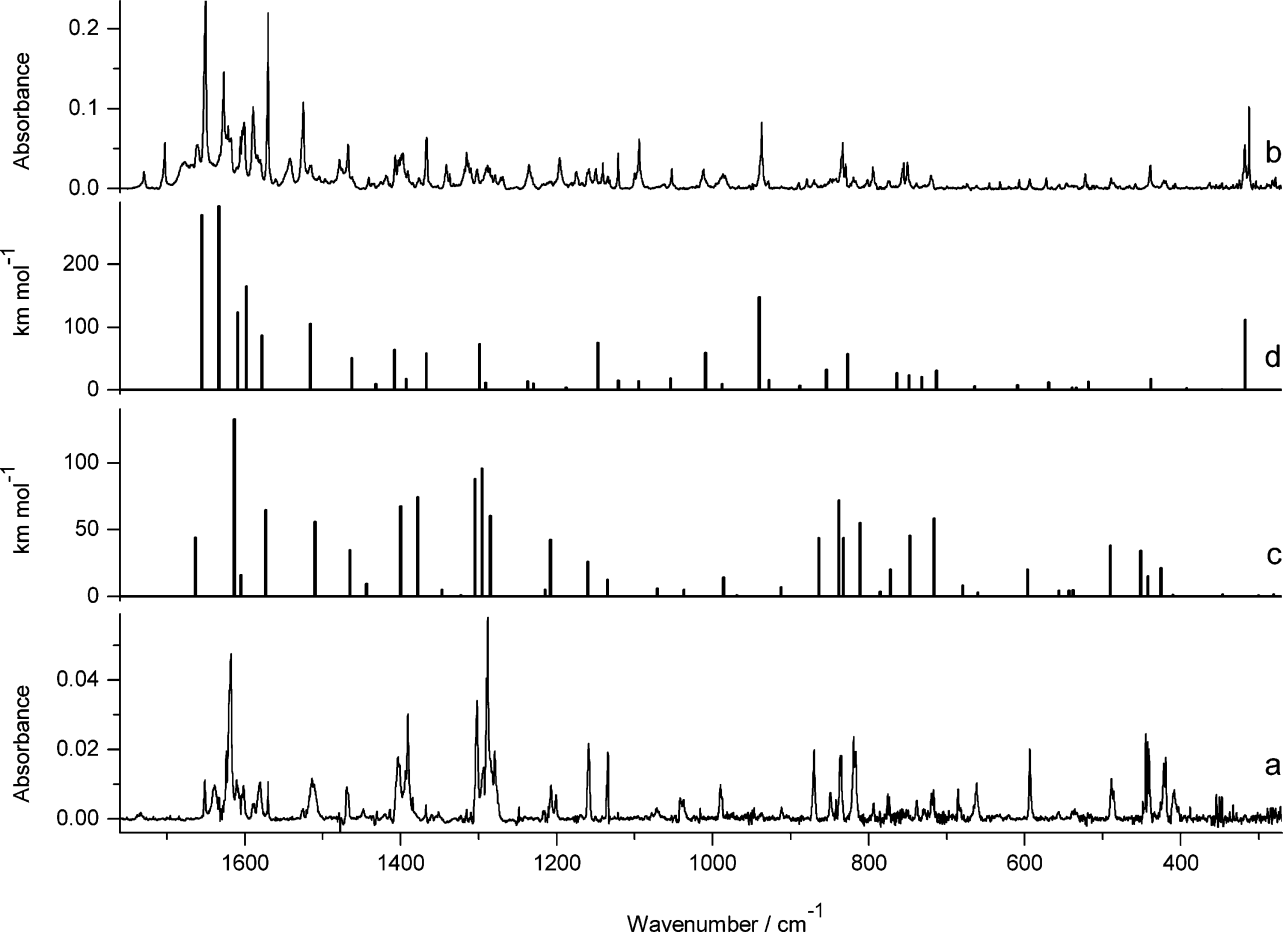
Infrared spectra of photoproducts generated upon UV irradiation of 7-hydroxyquinoline-8-aldoxime **4** isolated in an Ar matrix: (a) spectrum of the photoproduct generated upon 300 min of irradiation with UV (λ > 360 nm) light; (b) spectrum of the photoproduct generated upon 70 min of irradiation with UV (λ = 305 nm) light; compared with theoretical spectra, calculated at the DFT(B3LYP)/6-311++G(d,p) level, for the structures of plausible photoproducts presented in Scheme 3: (c) product **4**_**h2**_ resulting from the syn–anti isomerization within the hydroxy tautomer and (d) oxo product **4**_**o**_ resulting from the long-distance hydrogen-atom transfer.

Monomers of **4** isolated in an Ar matrix were irradiated with UV light of different wavelengths (Figure S7 in the Supporting Information). When long-wavelength UV (λ > 360 nm) light was applied, the initial (**4**_**h1**_) form of 7-hydroxyquinoline-8-aldoxime transformed into a product that can be identified as a result of the syn–anti isomerization within the aldoxime fragment (**4**_**h2**_ in Scheme 3). The infrared spectrum of the photoproduct generated after this irradiation is well reproduced by the spectrum theoretically predicted for **4**_**h2**_ (Figure 5a,c). In the infrared spectrum recorded after the UV (λ > 360 nm) irradiation, no bands which could be attributed to the oxo product **4**_**o**_ were observed.

**Figure 6:**
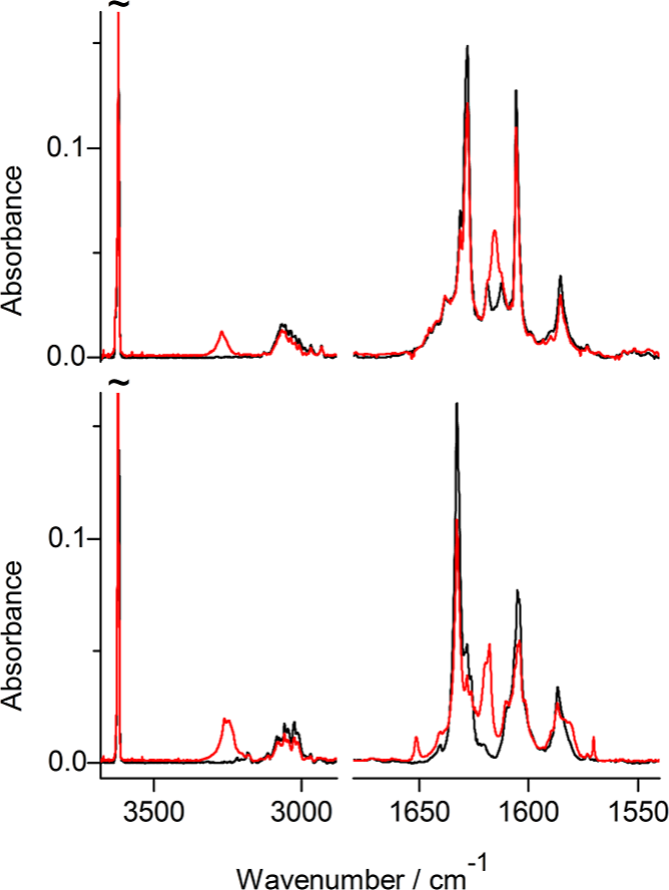
Effects of long-wavelength UV (λ > 360 nm) irradiation of 7-hydroxyquinoline-8-aldoxime **4** (lower panel) and 7-hydroxy-2-methylquinoline-8-aldoxime **5** (upper panel); spectra recorded after deposition of the matrices (black traces); spectra recorded after UV (λ > 360 nm) irradiation (red traces).

In the **4**_**h2**_ isomer of 7 hydroxyquinoline-8-aldoxime, the O–H group at position 7 of the quinoline ring is involved in an intramolecular hydrogen bond with the lone-electron pairs of the oxygen atom from the aldoxime fragment (Scheme 3). According to the DFT(B3LYP)/6-311++G(d,p) optimization of **4**_**h2**_ geometry, the structure of this part of the molecule (with the O–H···O intramolecular hydrogen bond) is considerably distorted from the planarity. Usually, the frequency of the stretching vibration of an O–H group engaged in the hydrogen bond is used as a measure of the strength of the H-bond interaction. The stronger the hydrogen bond, the lower the frequency of this vibration, with respect to the frequency of the stretching vibration of the O–H group not involved in any hydrogen bonding. The calculations performed for **4**_**h2**_ predict that the frequency of the stretching vibration of O–H engaged in the H-bond to the oxygen atom from the aldoxime group should be lower by 368 cm^–1^ than the frequency of the stretching vibration of free O–H from the aldoxime group. This theoretical value is in good agreement with the difference of the spectral positions of the experimentally observed bands. In the IR spectrum of the product generated in the syn–anti photoisomerization (**4**_**h2**_), the red-shifted νOH···O band appears at 3253 cm^–1^ (Figure 6). This wavenumber is lower by 367 cm^–1^ than the experimental spectral position (3620 cm^–1^) of the IR band due to the stretching vibration of the free O–H group belonging to the −C(H)=N–O–H fragment.

In the IR spectrum of the initial form **4**_**h1**_, the spectral shift of the band due to the stretching vibration of the O–H group directly involved in the intramolecular hydrogen bond is significantly larger. Theoretical calculations predict this shift to be as large as 476 cm^–1^. Correspondingly, in the experimental spectrum of the initial isomer of 7-hydroxyquinoline-8-aldoxime (**4**_**h1**_), the νOH···N band was observed at wavenumbers as low as 3100–2900 cm^–1^ (Figures 6 and S4 in the Supporting Information).

Not only the positions of the νOH···O and νOH···N bands in the infrared spectra of **4**_**h2**_ and **4**_**h1**_ forms of 7-hydroxyquinoline-8-aldoxime but also the shapes of these bands demonstrate that the hydrogen bond in **4**_**h2**_ is weaker than the hydrogen bond in **4**_**h1**_. In the experimental spectrum of the photoproduced isomer **4**_**h2**_, the band due to the stretching vibrations of the hydrogen-bonded O–H group (appearing at 3253 cm^–1^) is significantly narrower than the analogous broad band (at 3100–2900 cm^–1^) in the spectrum of the initial form **4**_**h1**_ (Figure 6).

Note that the syn–anti isomerization did not lead to important changes (greater than 5 cm^–1^) in the spectral position of the IR band due to the stretching vibration of the free O–H group belonging to the −C(H)=N–O–H fragment. In the spectra of both **4**_**h1**_ and **4**_**h2**_ isomers, the νOH band due to this group appears at wavenumbers close to 3620 cm^–1^ (Figure 6).

In a separate experiment, matrix-isolated 7-hydroxyquinoline-8-aldoxime **4** was irradiated with shorter-wavelength UV (λ = 305 nm) light. Upon such irradiation, a transformation to another product was observed. This product was identified as oxo tautomer **4**_**o**_ (Scheme 3 and Figure 5b,d). Hence, a photoinduced long-distance hydrogen-atom transfer occurred also for **4** (a compound with an aldoxime group playing the role of a crane), but this process required more excitation energy than the competing syn–anti photoisomerization within the aldoxime fragment.

**Figure 7:**
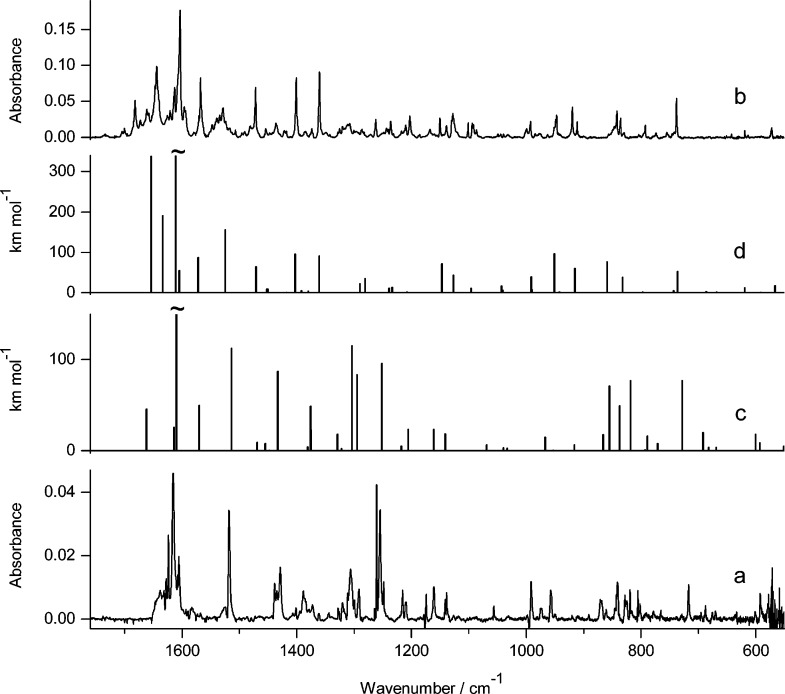
Infrared spectrum of photoproducts generated upon UV irradiation of 7-hydroxy-2-methylquinoline-8-aldoxime **5** isolated in an Ar matrix: (a) spectrum of the photoproduct generated upon 330 min of irradiation with UV (λ > 360 nm) light; (b) spectrum recorded after 120 min of exposure to UV (λ = 305 nm) light; compared with theoretical spectra, calculated at the DFT(B3LYP)/6-311++G(d,p) level, for the structures of plausible photoproducts presented in Scheme 3: (c) product **5**_**h2**_ resulting from the syn–anti isomerization within the hydroxy tautomer and (d) oxo product **5**_**o**_ resulting from the long-distance hydrogen-atom transfer.

UV-induced transformations, analogous to those observed for 7-hydroxyquinoline-8-aldoxime **4**, were also found for structurally similar 7-hydroxy-2-methylquinoline-8-aldoxime **5** (Scheme 3 and Figure S8 in the Supporting Information). Irradiation of matrix-isolated monomers of **5** with long-wavelength UV (λ > 360 nm) light led to a syn–anti isomerization involving rotation of the O–H group within the aldoxime fragment. The spectral indications of the product **5**_**h2**_ of the syn–anti isomerization were analogous to those observed for the nonmethylated parent compound (**4**, see Figures 6 and 7). Exposure of monomers of **5** to shorter-wavelength UV (λ = 305 nm) light resulted in conversion of the initial hydroxy tautomer **5**_**h1**_ to the oxo form **5**_**o**_ (Scheme 3). The same product **5**_**o**_ was generated upon UV (λ > 335 nm) irradiation of the matrix-isolated compound. The assignment of the photoproduct structure was based on a comparison of the experimental spectrum appearing upon UV excitation and the theoretical spectrum calculated for **5**_**o**_ (Figure 7b,d).

**Figure 8:**
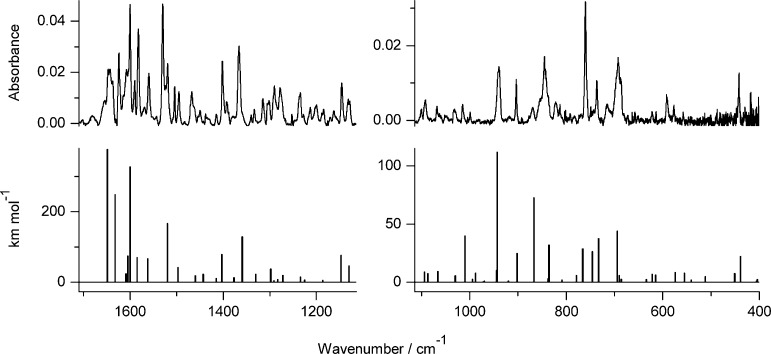
Infrared spectrum of photoproduct(s) generated upon 180 min of UV (λ > 295 nm) irradiation of 7-hydroxy-2-phenylquinoline-8-aldoxime **6** isolated in an Ar matrix, compared with theoretical spectra, calculated at the DFT(B3LYP)/6-311++G(d,p) level, for the oxo tautomer **6**_**o**_ (Scheme 4).

**Scheme 4:**
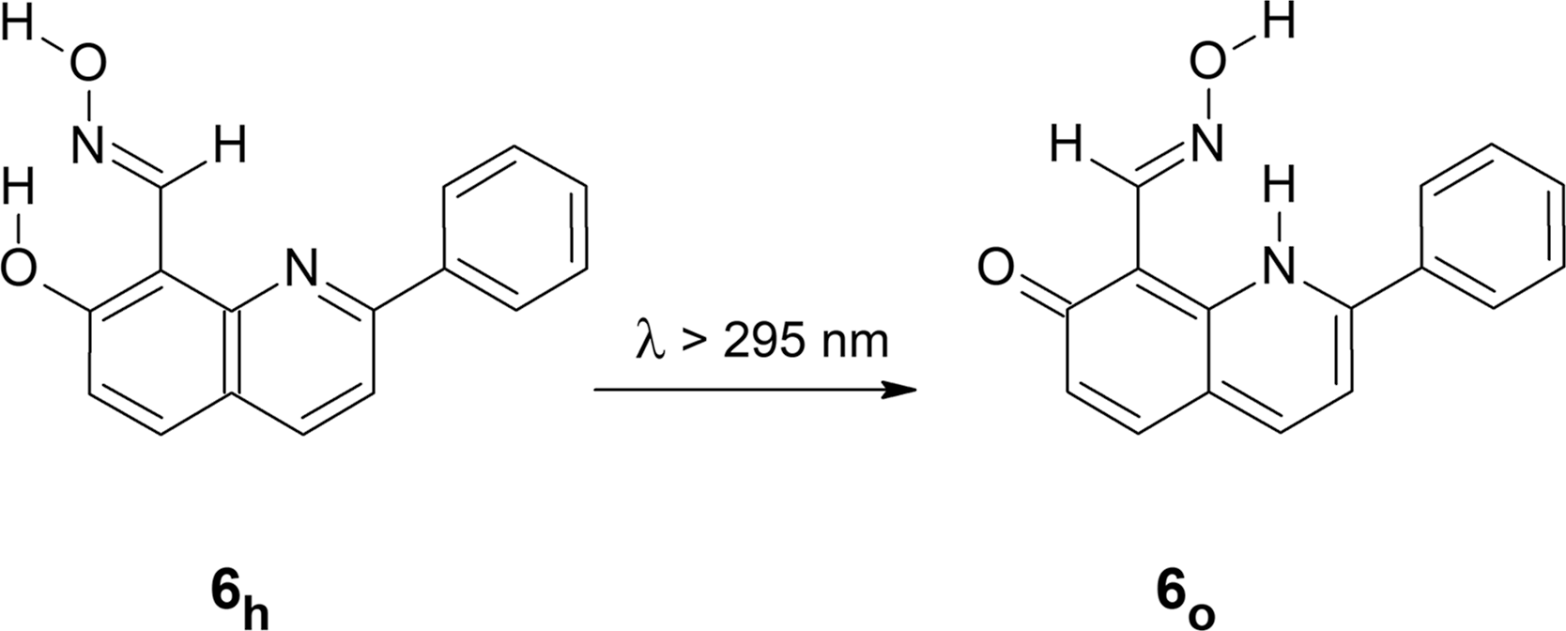
UV-Induced Long-Distance Hydrogen-Atom-Transfer Process in 7-Hydroxy-2-phenylquinoline-8-aldoxime (**6**)

The hydroxy → oxo phototautomeric conversion, involving long-distance hydrogen-atom transfer, was also investigated for 7-hydroxy-2-phenylquinoline-8-aldoxime **6** (Scheme 4 and Figure S9 in the Supporting Information). Comparison of the experimental IR spectrum of the product generated upon irradiation of matrix-isolated monomers of **6** with UV (λ > 295 nm) light with the spectrum theoretically calculated for the oxo tautomer **6**_**o**_ (Figure 8) suggests that photoinduced long-distance hydrogen atom transfer occurs also for this species.

## 5 Conclusions

UV-induced, long-distance hydrogen-atom transfer has been investigated for a number of compounds with a 7-hydroxyquinoline frame and the carbaldehyde or aldoxime groups serving as an intramolecular crane. The studies concerning 7-hydroxyquinoline-8-carbaldehyde **1** and 7-hydroxy-2-methylquinoline-8-carbaldehyde **2** very strongly corroborated the conclusion that photoinduced transfer of the hydrogen atom, from the O–H group attached at position 7 of the quinoline frame to the remote N1 atom, really occurs in 7-hydroxyquinoline derivatives with a carbaldehyde fragment at position 8. Investigations on 7-hydroxyquinoline derivatives with an aldoxime fragment at position 8 led to a somewhat more complicated picture of photochemical behavior. These studies revealed that compounds such as 7-hydroxyquinoline-8-aldoxime **4** or 7-hydroxy-2-methylquinoline-8-aldoxime **5**, isolated in low-temperature matrices and irradiated with UV (λ > 360 nm) light, undergo syn–anti isomerization involving rotation of the O–H group around the C=N double bond within the aldoxime fragment. Recently, similar photochemical behavior, that is, existence of two competitive channels: syn–anti isomerization and long-distance hydrogen transfer, was theoretically considered by Rode et al.^32^ for structurally similar compounds with a 7-hydroxyquinoline frame and a Schiff-base fragment attached at position 8 (Scheme 1e).

For 7-hydroxyquinoline-8-aldoxime **5**, 7-hydroxy-2-methylquinoline-8-aldoxime **6**, and 7-hydroxy-2-phenylquinoline-8-aldoxime **7**, a long-distance intramolecular hydrogen-atom transfer converting the hydroxy forms of these compounds into the oxo tautomers was found to occur upon irradiation with shorter UV wavelengths (λ = 305 nm, λ > 335 nm, or λ > 295 nm). These experimental findings demonstrate that photoinduced long-distance hydrogen-atom transfer processes can also occur with involvement of the aldoxime group acting as the intramolecular crane.

## Supporting Information Available

Spectral identification of the isomeric forms of studied compounds trapped into low-temperature argon matrices based on the comparison of the IR spectra with the spectra simulated at the DFT(B3LYP)/6-311++G(d,p) level of theory; IR spectra of the studied compounds in the region 3700–2500 cm^–1^; and characteristic of the stretching vibrations of the O–H groups (PDF)
