# Supplementary material for: Photoinduced Long-Distance Hydrogen-Atom Transfer in Molecules with a 7-Hydroxyquinoline Frame and a Carbaldehyde or Aldoxime Group as the Intramolecular Hydrogen Transporting Crane
Source: J Phys Chem A. 2023 Apr 3;127(14):3104–13. doi: 10.1021/acs.jpca.3c00170 (PMC10108369; doi:10.1021/acs.jpca.3c00170)
Supplement: Supplementary file 1 — jp3c00170_si_001.pdf [file jp3c00170_si_001.pdf]

Supporting Information

Photoinduced Long-Distance Hydrogen-Atom Transfer in Molecules with 7-Hydroxyquinoline Frame and Carbaldehyde or Aldoxime Group as Intramolecular Hydrogen Transporting Crane

Leszek Lapinski, Hanna Rostkowska, Jacek Nowacki and Maciej J. Nowak

Institute of Physics, Polish Academy of Sciences,
Al. Lotników 32/46, 02-668 Warsaw, Poland

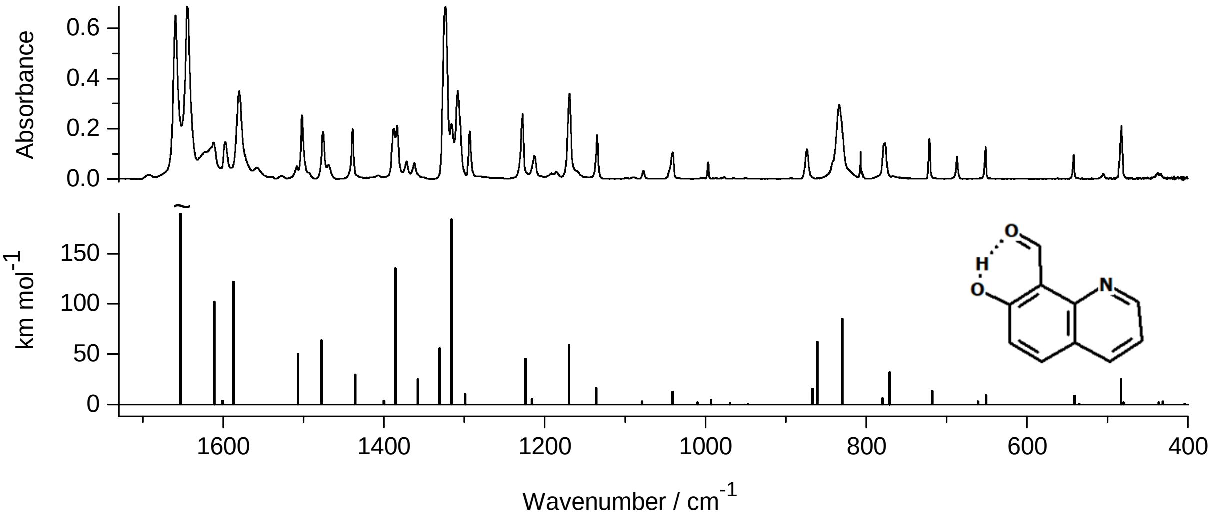

Figure S1 Infrared spectrum of 7-hydroxyquinoline-8-carbaldehyde **1** isolated in an Ar matrix compared with theoretical spectrum, calculated at the DFT(B3LYP)/6-311++G(d,p) level for the hydroxy tautomer (**1_h**) of the compound.

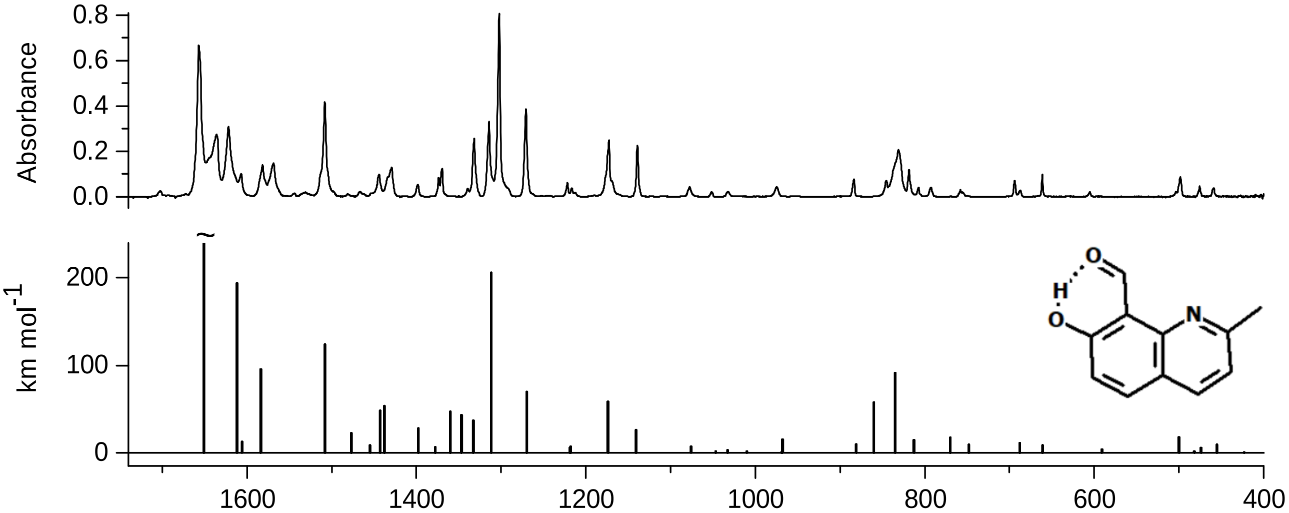

Figure S2 Infrared spectrum of 7-hydroxy-2-methylquinoline-8-carbaldehyde **2** isolated in an Ar matrix compared with theoretical spectrum, calculated at the DFT(B3LYP)/6-311++G(d,p) level for the hydroxy tautomer (**2_h**) of the compound.

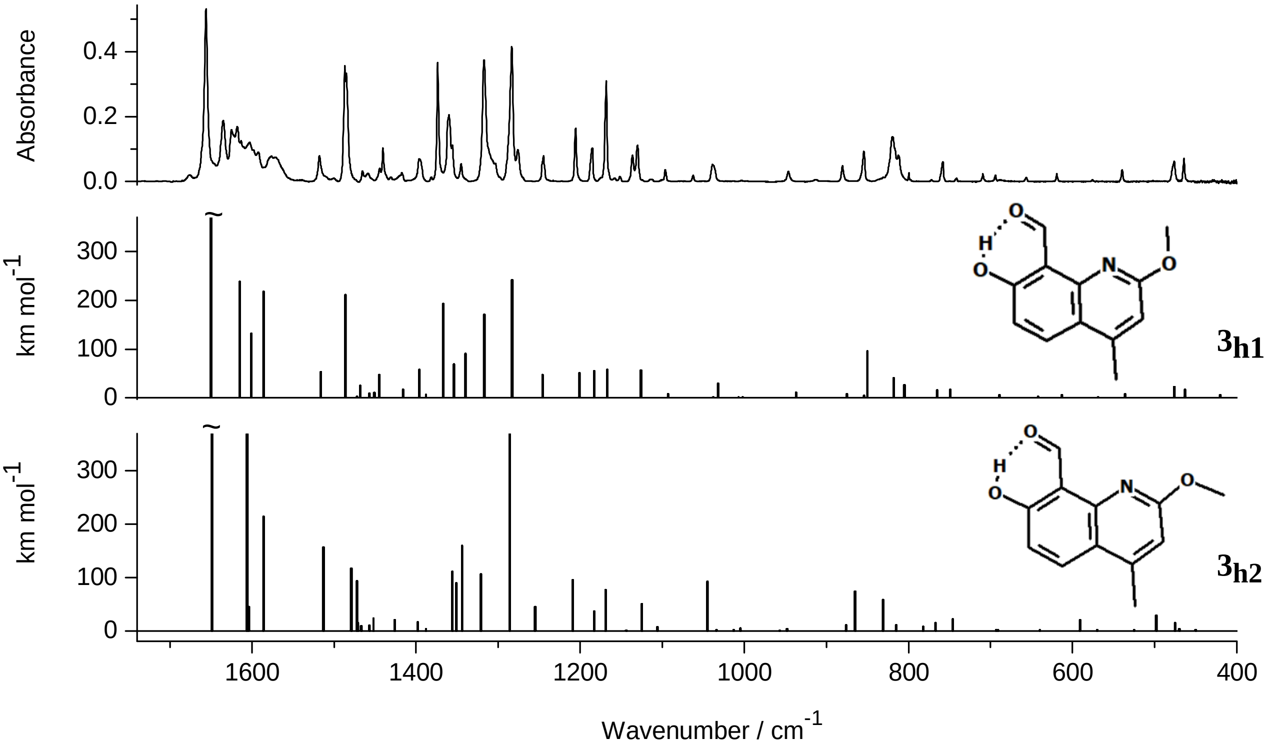

Figure S3 Infrared spectrum of 7-hydroxy-2-methoxy-4-methylquinoline-8-carbaldehyde **3** isolated in an Ar matrix compared with theoretical spectra calculated at the DFT(B3LYP)/6-311++G(d,p) level, for two isomers (**3_{h1}** and **3_{h2}**) of the hydroxy tautomer of the compound.

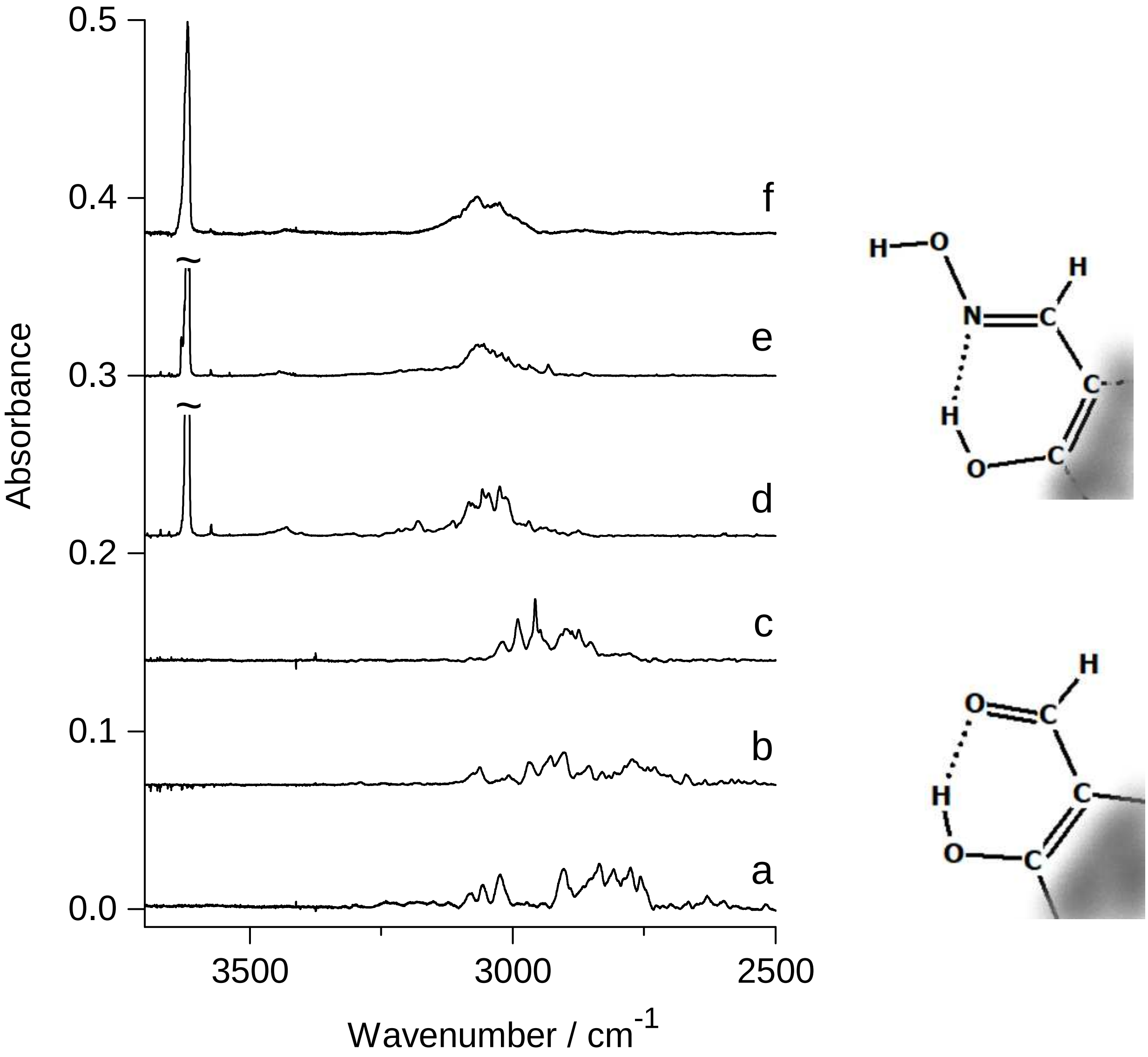

Figure S4 Fragments of the IR spectra of: (a) 7-hydroxyquinoline-8-carbaldehyde,**1**; (b) 7-hydroxy-2-methylquinoline-8-carbaldehyde, **2**; (c) 7-hydroxy-2-methoxy-4-methylquinoline-8-carbaldehyde, **3**; (d) 7-hydroxyquinoline-8-aldoxime, **4**; (e) 7-hydroxy-2-methylquinoline-8-aldoxime, **5**; (f) 7-hydroxy-2-phenylquinoline-8-aldoxime, **6**, all isolated in Ar matrices.

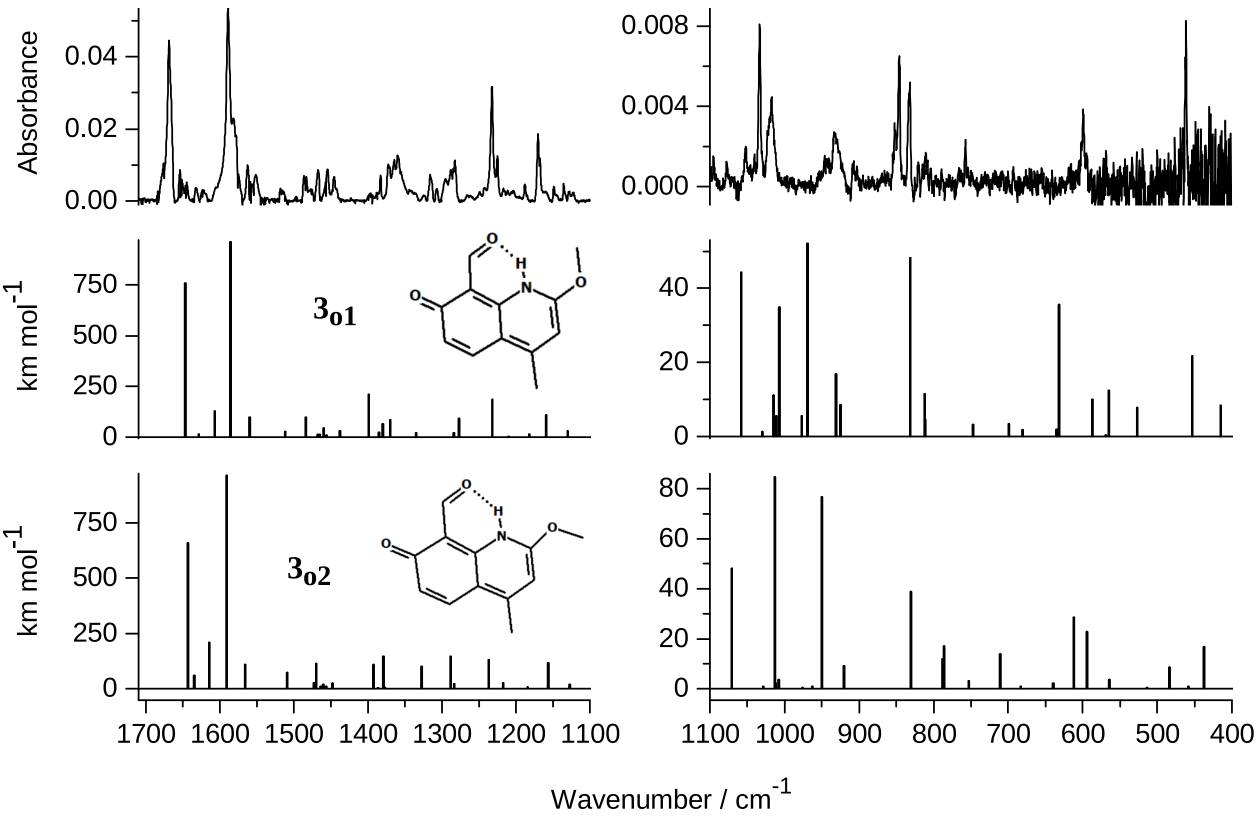

Figure S5 Infrared spectrum of photoproduct(s) generated upon 140 min of UV (λ > 270 nm) irradiation of 7-hydroxy-2-methoxy-4-methylquinoline-8-carbaldehyde **3** isolated in an Ar matrix compared with theoretical spectra calculated at the DFT(B3LYP)/6-311++G(d,p) level, for two isomers (**3$_{o1}$** and **3$_{o2}$**) of the oxo tautomer of the compound.

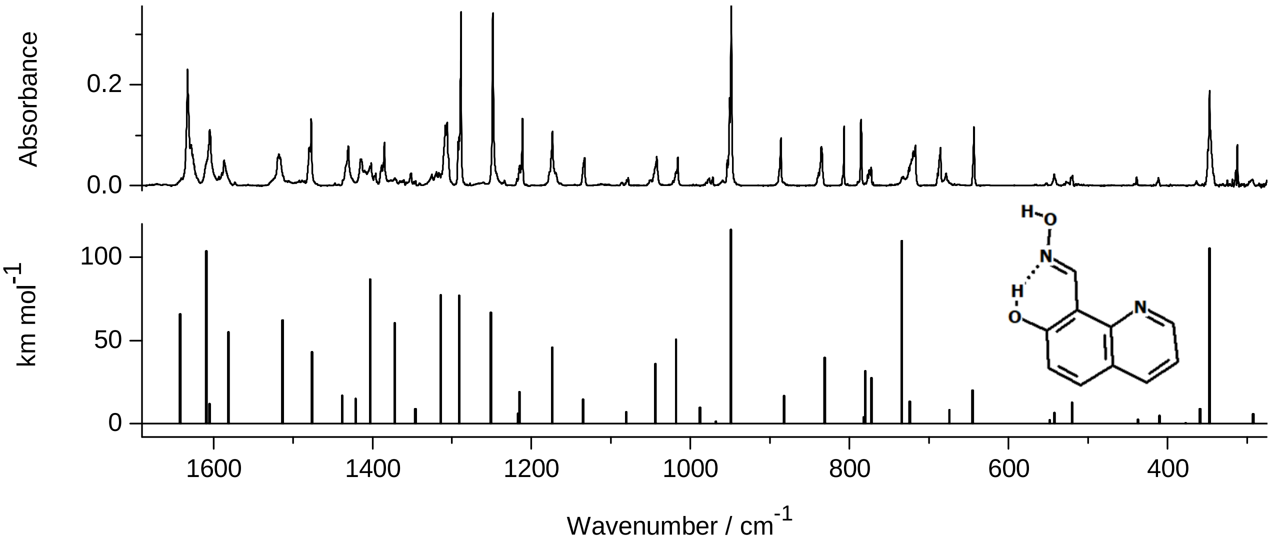

Figure S6 Infrared spectrum of 7-hydroxyquinoline-8-aldoxime **4** isolated in an Ar matrix compared with theoretical spectrum calculated at the DFT(B3LYP)/6-311++G(d,p) level, for the hydroxy tautomer (**4**$_{\mathbf{h1}}$) of the compound.

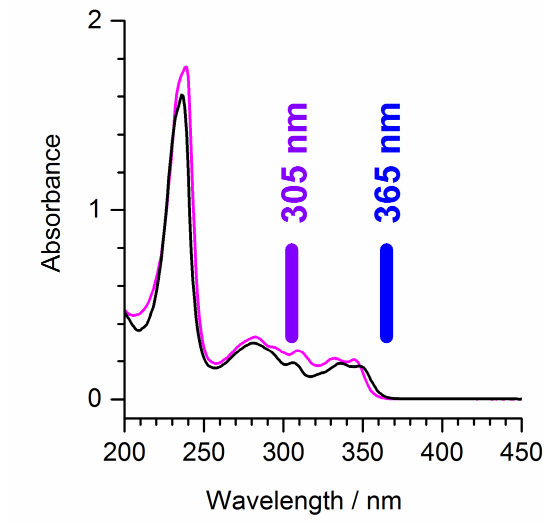

Figure S7 UV absorption spectra of 7-hydroxyquinoline-8-aldoxime (**4**, black) and 7-hydroxy-2-methylquinoline-8-aldoxime (**5**, magenta) dissolved in acetonitrile.

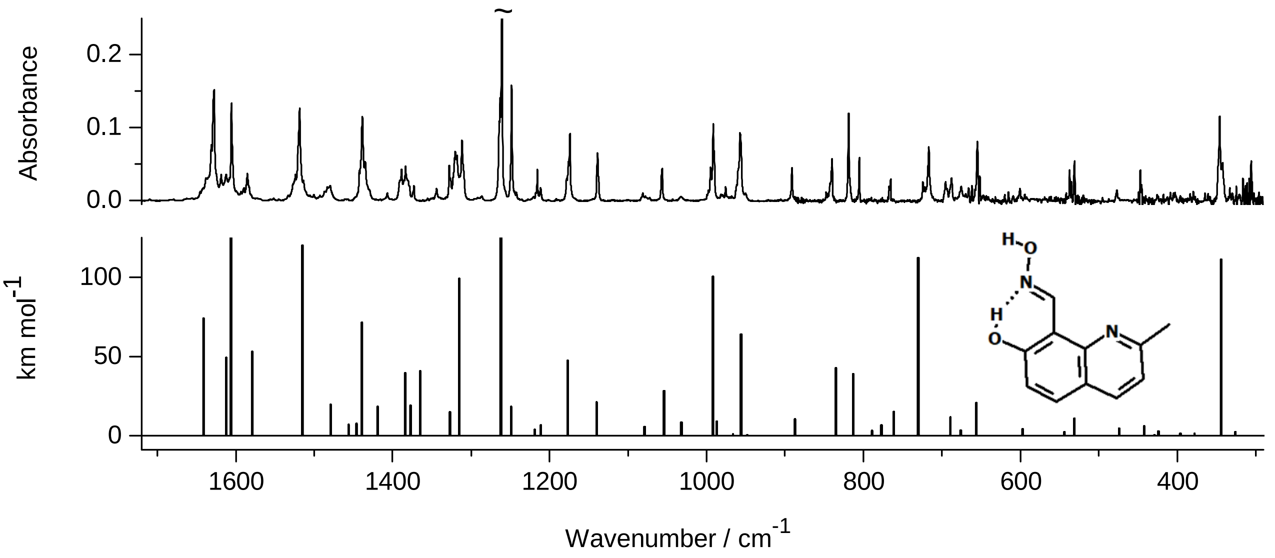

Figure **S8** Infrared spectrum of 7-hydroxy-2-methylquinoline-8-aldoxime **5** isolated in an Ar matrix compared with theoretical spectrum, calculated at the DFT(B3LYP)/6-311++G(d,p) level, for the hydroxy tautomer ($\mathbf{5_{h1}}$) of the compound.

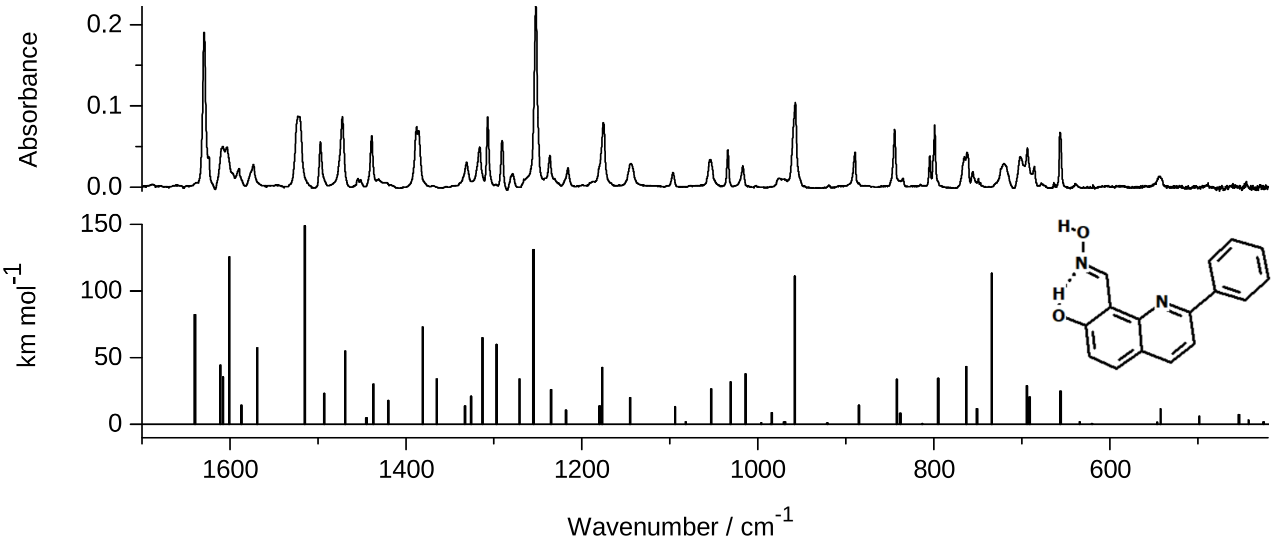

Figure S9 Infrared spectrum of 7-hydroxy-2-phenylquinoline-8-aldoxime **6** isolated in an Ar matrix compared with theoretical spectrum calculated at the DFT(B3LYP)/6-311++G(d,p) level, for the hydroxy tautomer ($\mathbf{6_{h}}$) of the compound.
